# Association between preoperative neurocognitive status and IDH1 mutation status in high-grade gliomas

**DOI:** 10.1093/nop/npac077

**Published:** 2022-09-30

**Authors:** Evangelia Liouta, Aristotelis V Kalyvas, Spyridon Komaitis, Evangelos Drosos, Christos Koutsarnakis, Juan M García-Gómez, Javier Juan-Albarracín, Vasileios Katsaros, Theodosis Kalamatianos, Theodoros Argyrakos, George Stranjalis

**Affiliations:** Department of Neurosurgery, National and Kapodistrian University of Athens, Evangelismos Hospital, Athens, Greece; Hellenic Center for Neurosurgical Research “Prof. Petros Kokkalis”, Athens, Greece; Department of Neurosurgery, National and Kapodistrian University of Athens, Evangelismos Hospital, Athens, Greece; Division of Neurosurgery, Toronto Western Hospital, University Health Network, University of Toronto, Toronto, Ontario, Canada; Athens Microneurosurgery Laboratory, Athens, Greece; Department of Neurosurgery, National and Kapodistrian University of Athens, Evangelismos Hospital, Athens, Greece; Athens Microneurosurgery Laboratory, Athens, Greece; Department of Neurosurgery, National and Kapodistrian University of Athens, Evangelismos Hospital, Athens, Greece; Athens Microneurosurgery Laboratory, Athens, Greece; Department of Neurosurgery, National and Kapodistrian University of Athens, Evangelismos Hospital, Athens, Greece; Athens Microneurosurgery Laboratory, Athens, Greece; Grupo de Informática Biomédica (IBIME), Instituto de Aplicaciones de las Tecnologías de la Información y de las Comunicaciones Avanzadas (ITACA), Universitat Politècnica de València, Valencia, Spain; Grupo de Informática Biomédica (IBIME), Instituto de Aplicaciones de las Tecnologías de la Información y de las Comunicaciones Avanzadas (ITACA), Universitat Politècnica de València, Valencia, Spain; Department of Radiology, General Anti-Cancer and Oncological Hospital of Athens “St. Savvas”, Athens, Greece; Department of Neurosurgery, National and Kapodistrian University of Athens, Evangelismos Hospital, Athens, Greece; Hellenic Center for Neurosurgical Research “Prof. Petros Kokkalis”, Athens, Greece; Department of Pathology, Evangelismos Hospital, Athens, Greece; Department of Neurosurgery, National and Kapodistrian University of Athens, Evangelismos Hospital, Athens, Greece; Hellenic Center for Neurosurgical Research “Prof. Petros Kokkalis”, Athens, Greece; Athens Microneurosurgery Laboratory, Athens, Greece

**Keywords:** cognition, executive functions, high-grade gliomas, IDH1 mutation, neuropsychological testing

## Abstract

**Background:**

High-grade glioma (HGG) patients present with variable impairment in neurocognitive function (NCF). Based on that, isocitrate dehydrogenase 1 (IDH1) wild-type HGGs are more aggressive than IDH1 mutant-type ones, we hypothesized that patients with IDH1 wild-type HGG would exhibit more severe NCF deficits than their IDH1 mutant counterparts.

**Methods:**

NCF was assessed by Mini Mental Status Exam (MMSE), Trail Making Test (TMT), Digit Span (DS), and Controlled Word Association Test (COWAT) tests in 147 HGG patients preoperatively.

**Results:**

Analyses between IDH1 groups revealed a significant difference on MMSE concentration component (*p* ≤ .01), DS (*p* ≤ .01), TMTB (*p* ≤ .01), and COWAT (*p* ≤ .01) scores, with the IDH1 wild group performing worse than the IDH1 mutant one. Age and tumor volume were inversely correlated with MMSE concentration component (*r* = −4.78, *p* < .01), and with MMSE concentration (*r* = −.401, *p* < .01), TMTB (*r* = −.328, *p* < .01), and COWAT phonemic scores (*r* = −.599, *p* < .01), respectively, but only for the IDH1 wild-type group. Analyses between age-matched subsamples of IDH1 groups revealed no age effect on NCF. Tumor grade showed nonsignificance on NCF (*p* > .05) between the 2 IDH1 mutation subgroups of grade IV tumor patients. On the contrary, grade III group showed a significant difference in TMTB (*p* < .01) and DS backwards (*p* < .01) between IDH1 subgroups, with the mutant one outperforming the IDH1 wild one.

**Conclusions:**

Our findings indicate that IDH1 wild-type HGG patients present greater NCF impairment, in executive functions particularly, compared to IDH1 mutant ones, suggesting that tumor growth kinetics may play a more profound role than other tumor and demographic parameters in clinical NCF of HGG patients.

In the Central Nervous System (CNS) World Health Organization (WHO) 2016 tumor classification system, genetic markers were added as new parameters according to which brain gliomas are now categorized in a more tailored fashion. These genetic markers seem to have a stronger prognostic value for survival with the most promising being the mutation of the isocitrate dehydrogenase 1 (IDH1) gene.^1–3^ IDH1 mutation is a metabolic enzyme in the glucolytic pathway that catalyzes the oxidative decarboxylation of isocitrate to 2-oxoglutarate providing cellular protection from oxidative stress.^4,5^

Regarding the high-grade gliomas (HGGs), IDH1 mutation is found in the majority (50% to over 80% range) of grade III gliomas^1,2,6–9^ and in the minority (5%–13%) of grade IV glioblastoma (GBM), respectively.^1,2,7–9^ Accumulative evidence supports that IDH1 mutation in malignant gliomas provides a prognostic value in overall survival and progression-free survival^1–3^ with patients harboring grade III astrocytoma or grade IV GBM with the absence of IDH1 mutation (wild type) to exhibit worse prognosis than their IDH1 mutant counterparts; even more interestingly, IDH1 wild-type anaplastic astrocytomas appear to have a poorer prognosis than IDH1 mutant-type GBMs.^10^

Such heterogeneity in survival may mirror the differences in tumor growth kinetics between IDH1 mutant and wild-type gliomas, with wild-type gliomas showing a faster rate cell proliferation than the mutant ones.^11^ Differences in HGG proliferation may also play a substantial role in the heterogeneity of patients’ symptoms at admission. This can be attributed to neuroplasticity, that is, the brain’s potential for cerebral reorganization^12^ with IDH1 wild-type HGG producing more frequent and severe clinical symptoms due to limited neuroplasticity caused by their rapid growth pattern. As neurocognitive impairment consists of a core domain of the clinical symptoms that tumor patients show,^13,14^ one would assume that IDH1 wild-type HGG would induce more severe neurocognitive deficits compared to their IDH1 mutant-type counterparts. Literature on the relationship between IDH1 subtypes of HGG and neurocognitive function (NCF) is scarce.^15^ Taken all the above into consideration, the present study sought to investigate the association between NCF and IDH1 mutation status in a large cohort of patients with HGG planned for surgical treatment. According to our hypothesis, we expected that NCF of IDH1 wild-type HGG patients would be more severely affected than their IDH1 mutant-type HGG counterparts.

## Materials and Methods

### Participants

One hundred and sixty patients with untreated supratentorial HGGs (grade III anaplastic astrocytoma and grade IV glioblastoma) undergoing surgical treatment at the Neurosurgical Clinic of University of Athens, “Evangelismos” Hospital between 2015 and 2019 were neuropsychologically assessed prior to surgery by an experienced neuropsychologist (first author) as part of our clinical protocol. After excluding patients with (a) severe aphasia hindering communication (*n* = 6), (b) other concomitant brain disorders (*n* = 3) and (c) patients under psychotropic medication at the time of diagnosis (*n* = 4), we ended up with 147 patients participating in the present study. All patients were operated by the same neurosurgeon (last author). The majority of patients were under corticosteroid treatment when evaluated. Informed consent was obtained from patients prior to commencing the procedure. The present study was conducted according to the Declaration of Helsinki.

### Spatial Parameters

Preoperative brain magnetic resonance imaging (MRI) scans were obtained 7.3 ± 2.5 days before the preoperative neurocognitive assessment. MRI scans were reviewed and the tumor location was categorized according to the cerebral lobe the lesion occupied (frontal, parietal, temporal [including insula], and occipital). Tumors that occupied more than 1 lobe were categorized according to the tumor’s largest volume location. MRI volume calculations were performed using the ONCO habitats software system (https://www.oncohabitats.upv.es).^16^ For MRI volumetric segmentations, preprocessing was first employed using the following scheme for our data: (1) denoising, (2) skull stripping, (3) bias field correction, and (4) super-resolution. Following preprocessing, the methods of feature extraction and dimensionality reduction and unsupervised voxel classification were employed. Finally, the following method to automatically isolate tumor classes was used: (1) identify and remove white matter, gray matter, and cerebrospinal fluid classes; (2) remove outlier classes; and (3) merge classes by statistical distribution similarities. For more information, see https://www.oncohabitats.upv.es16. Lesion volumetry was conducted in a blind fashion to molecular stratification.

### Cognitive Assessment

All patients were assessed for global neurocognitive status with the Mini Mental Status Examination (MMSE) prior to surgery with a mean of 6.7 ± 3.1 days. MMSE is a well-established index of global cognition; it begins by assessing orientation to time and place, memory coding and recall, concentration and arithmetic subtraction, language by object naming, words’ repetition and comprehension of written words, upper limb praxis by compliance with a three-step command, and visuospatial processing-constructional praxis by copying a drawing.^17^ The maximal score for the entire MMSE is 30 points.

Patients were also assessed for executive functions, known to be one of the most sensitive and affected cognitive domains in tumor patients.^18^ Assessment consisted of a standardized neuropsychological battery of the following tests: Wechsler Adult Intelligence Scale-III Digit Span (DS) test^19^ (the forward and backward subtests measuring short-term and working memory, respectively); Trail Making Test (TMT) Parts A and B,^20^ measuring speed processing and complex attention, respectively; Controlled Oral Word Association Test (COWAT)^21^—including phonemic and category subsets—a verbal fluency test that combines assessment of language and executive function. COWAT was administered only to patients with dominant hemisphere lesions. The dominant hemisphere was determined by the combination of the following data: (a) Handedness Edinburgh Inventory, (b) clinical symptoms, and (c) fMRI for language laterality and localization.

### Histopathology

For the majority of patients, a histopathological diagnosis was determined according to WHO 2016 criteria. We should clarify that only IDH1R132 was included in the analysis. For the purpose of the study, we only analyzed IDH1 and its relationship with cognitive status. Regarding IDH1, for all patients, there was available paraffin-embedded tissue scored for it. Due to the IDH2 mutation rarity in astrocytic gliomas,^7^ IDH2 was not scored. IDH1R132 immunostaining took place following routine procedures. Briefly, formalin-fixed, paraffin-embedded tissue sections (4 mm thick) were cut and dried for 24 h at 37°C on a hot plate. Slides were deparaffinized in xylene and rehydrated in graded ethanols. Immunohistochemical staining for IDH1R132H (clone H09, Dianova, Germany) was performed on an automated Autostainer Link 48 immunohistochemical slide stainer (DAKO) and visualized using EnVision Flex+ High pH system (DAKO). Pilocytic astrocytoma sections were used as negative controls for IDH1R132H immunostaining. A binomial classification of “positive” or “negative” was used for the IDH1R132H immunostain, according to the presence or absence of strong cytoplasmic staining in any number of neoplastic cells.^22^

### Statistical Analysis

Statistical analyses were performed with SPSS 22.0 (IBM). Descriptive statistics for demographics (age, gender, and education), tumor (grade, laterality, localization, and volume), and NCF (MMSE, DS, TMT, and COWAT) parameters were calculated as means and standard deviations for continuous variables or frequencies and percentages for dichotomous variables. Data were not normally distributed according to Kolmogorof–Smirnov normality test (*p* < .05); therefore, nonparametric tests were employed. Nonparametric-independent samples *t-*tests (Welch’s test) and chi-square goodness-of-fit tests were used to compare differences in demographics and tumor characteristics between IDH1 wild-type and IDH1 mutant-type groups.

MMSE raw scores (total and subtotals for each component), DS forward and backward, TMT A and B, and COWAT phonemic and semantic scores were treated as continuous variables and compared between IDH1 mutation groups with nonparametric-independent 2-sample *t-*test. For DS, TMT, and COWAT analyses, we used *z*-converted scores according to patients’ age and education.

Associations among NCF tests, demographics, and lesion characteristics were calculated with Spearman’s nonparametric correlations for continuous variables or chi-square goodness-of-fit tests for dichotomous ones and where appropriate with nonparametric-independent 2-sample *t-*test (Welch’s test) or Analysis of Variance (ANOVA) Kruskal–Wallis test. ANCOVA analyses with IDH1 mutation status as an independent factor and cognitive measures as dependent variables with demographics and tumor parameters as covariates were also conducted. Two-sided tests were used with a significance level of *p* ≤ .05 with Bonferroni corrections applied where multiple comparisons were employed.

## Results

### Demographics and Tumor Characteristics

#### Total sample.—

Mean age of patients was 55.7 years ± 14.3 (range = 23–82) with male/female ratio *n* = 90(61.2%)/*n* = 57(38.8%) and education mean = 12 years ± 2.2 (range = 6–16). As per tumor localization in the total sample, 43.5% of patients harbored a frontal glioma, 40.8% a temporal glioma, 15% a parietal one, and 0.7% an occipital one. Regarding laterality, 60.5% of gliomas were located in the left hemisphere. Concerning tumor pathology, 117 (79.5%) of patients were diagnosed with GBM (grade IV) and the rest with anaplastic astrocytoma (grade III). Lesion volume mean was 91.2 cm^3^ ± 54.4 (range = 2.9–220).

#### IDH1 mutation status groups.

—Gender ratio was not statistically different (*p* > .05) between IDH1 mutant and wild-type groups. Similarly, there was no statistical difference (*p* > .05) in educational level between IDH1 mutant- and wild-type groups. In line with the current literature, patients’ age at diagnosis showed a statistically significant difference (*p* < .05) with the IDH1 mutant group having a younger mean age than the IDH1 wild one.^23^ Concerning pathology, the majority of glioblastomas were IDH1 wild type, while the majority of anaplastic astrocytomas were IDH1 mutant, as expected. Regarding the tumor laterality, there was no statistically significant difference (*p* > .05) between the 2 IDH1 mutation groups. Concerning localization, the majority of gliomas were located in the frontal and temporal lobes of both groups. Finally, the tumor volume mean was similar across IDH1 mutation groups (*p* > .05). Demographical and tumor parameters for IDH1 mutation groups are described in Table 1.

**Table 1.** Demographics and tumor characteristics for both IDH1 mutation status groups

	IDH1 wild type	IDH1 mutant	Comparison *p*-value
Age (years)			
Mean (SD)	58.8 (12.2)	40.7 (14.5)	.035*
Range	23–75	23–82	
Gender			
Male *N* (%)	76 (64.9)	17 (56.6)	.355
Education (years)			
Mean (SD)	11.8 (2.1)	12.6 (2.4)	.122
Tumor characteristics			
Grade *N* (%)			<.01^**^
IV	109 (93)	8 (7)	
III	8 (26.7)	22 (73.3)	
Laterality			
Left *N* (%)	74 (63)	15 (50)	.561
Location			.152
Frontal	46 (39.3)	16 (53.3)	
Temporal	50 (42.7)	10 (33.3)	
Parietal	20 (17)	4 (13.3)	
Occipital	1 (0.85)	0 (0)	
Volume			
Mean (SD)	88.2 (55.9)	100.6 (49.3)	.368

*Statistical significance at level *p* = .05.

^**^Statistical significance at level *p* = .01.

### Neurocognitive Results

#### Neuropsychological tests and IDH1 mutation status.

—In the total number of patients, the MMSE mean score was 23.8 ± 5.3 (range 4–30). Comparison of the continuous variable of MMSE total score between IDH1 subgroups showed a statistically significant difference (*p* ≤ .05, mean difference= 3.7, 95% CI= 2.1–5.4), with the IDH1 wild group (23.1 ± 5.4) performing worse than the IDH1 mutant one (26.9 ± 3.3). After multiple comparisons correction, however, this difference did not survive the statistical significance.

Comparisons of MMSE subscores between IDH1 subgroups showed a statistically significant difference for orientation (*p* < .05) and concentration-calculation (*p* ≤ .01) components with the IDH1 wild group performing worse than the IDH1 mutant one. After multiple corrections, however, only the concentration-calculation subscale survived statistically significance (*p* ≤ .01) (see Table 2).

**Table 2.** Performance on MMSE components by IDH1 mutation status

MMSE component	IDH1 status groups		*p*-Value
	IDH1 mutant	IDH1 wild type	
Orientation	9.10(2.4)	6.98(3.6)	.046
Immediate memory	2.81(0.3)	2.58(0.8)	.131
Concentration/calculation	4.72(1.1)	3.47(0.5)	<.01*
Delayed recall	2.57(0.3)	2.28(0.3)	.097
Naming	1.90(0.2)	1.76(0.4)	.233
Verbal repetition	0.82(0.4)	0.73(0.3)	.391
Verbal comprehension	2.71(0.4)	2.40(0.8)	.081
Writing	0.93(0.5)	0.81(0.3)	.114
Reading a sentence	0.89(0.3)	0.90(0.2)	.368
Copying DESIGN	0.94(0.3)	0.87(0.5)	.225

*p-Values are significant at the .01 level adjusted for multiple comparisons with Bonferroni correction. Data are presented as raw scores means (SD).

Regarding the executive function tests, DS forward subscale score was comparable between IDH1 mutation groups (*p* ≥ .5), while there was a statistically significant difference (*p* ≤ .01) in DS backward subscale with IDH1 mutant group to outperform the IDH1 wild one. Statistically significant difference was also found between the two groups in TMT A (*p* ≤ .05) and B (*p* ≤ .01) and in COWAT phonemic subset (*p* ≤ .01) with IDH1 wild group to perform poorer than the mutant one. Semantic subscale, on the other hand, was not found significantly different (*p* > 0.5) between the two groups. After Bonferroni correction, the test-survived statistical significance was DS backwards (*p* ≤ .01), TMT B (*p* ≤ .01), and COWAT phonemic subset (*p* < .01) (see Table 3). The executive function measures of DS, TMT, and COWAT were also treated as categorical values with statistical analysis to yield similar results with the ones employing continuous variables (see Table 4).

**Table 3.** Performance on executive functions assessment by IDH1 mutation status

Test	IDH1 status		*p*-Value
	IDH1 mutant	IDH1 wild type	
TMT A	−0.23(0.97)	−0.57(1.74)	.075
TMT B	−0.67(1.43)	−1.76(2.21)	<.01*
DS forwards	0.33(0.46)	−0.19(0.62)	.057
DS backwards	−0.17 (0.44)	−1.12(1.01)	<.01*
COWAT phonemic	0.26(0.72)	−1.34(1.22)	.01*
COWAT semantic	0.17(0.55)	−0.63(0.97)	.246

Data are presented as *z*-scores means (SD).

*p-Values are significant at the .01 level adjusted for multiple comparisons with Bonferroni correction.

COWAT, Controlled Word Association Test; DS, Digit Span; TMT, Trail Making A.

**Table 4.** Frequency of neurocognitive impairment by IDH1 status

Test	IDH1mutant	IDH1 wild type	p-Value
	*N* (%)	*N* (%)	
TMT A	4(13.3)	25(21.3)	.11
TMT B	8(28.6)	82(70)	<.01*
DS forwards	4(13.3)	22(18,8)	.26
DS backwards	3(10)	55(47)	<.01*
COWAT phonemic	8(26.6)	67(57.2)	<.01*
COWAT semantic	7(23.3)	40(34.1)	.37

Impairment defined as a *z*-score ≤−1.5 for all measures.

*Significant difference between groups, *p* ≤ .01; chi-square goodness-of-fit tests.

#### Neuropsychological tests and demographics.

—In regards with the whole sample, DS backwards, TMT B, and COWAT phonemic subset scores distributed comparably between male and female patients (*p* = .43, *p* = .22, and *p* = .09, respectively) and they were not significantly correlated with education(*r* = 0.24/*p* = .455, *r* = −1.32/ *p* = .620, and *r* = −0.9/*p* = .22, respectively) or age (*r* = −0.13/ *p* = .747, *r* = 0.68/ *p* = .620, and *r* =.027/*p* = .518, respectively). The distribution of MMSE concentration-calculation subscale mean performance was comparable (*p* = .879) between males and females, it was not significantly (*r* = −.011, *p* = .756) correlated with patients’ education level, but it showed a statistically significant inverse correlation (*r* = −4.59, *p* < .01) with patients’ age.

When we split the data according to IDH1 status, there was no statistically significant difference (*p* > .05) in the MMSE concentration-calculation subscale mean between males and females neither for IDH1 mutant group nor for IDH1 wild one. Education was not significantly correlated with MMSE concentration-calculation performance neither for the IDH1 mutant (*r* = .012, *p* = .654) nor for the IDH1 wild group (*r* = −.15, *p* = .323). Age, on the other hand, was inversely correlated with it; however, only for the IDH1 wild-type group (*r* = −4.78, *p* < 0.01) and not for the IDH1 mutant one (*r* = −.121, *p* > 0.05) MMSE and IDH1 mutation status in young patients.

As the mean age of IDH1 wild group was significantly (<.05) higher than that of IDH1 mutant group, and age was significantly correlated with MMSE performance in the IDH1 wild group, we additionally run comparison analyses in a subset of our sample with patients’ age under 50 years as an inclusion criterion, in order to control for the confounding variable of age. Accordingly, we ended up with a total sample of 52 patients (mutant group *n*= 24/ wild group *n*=28). Levene’s test for equality of variances between the IDH1 groups was found significant (*p* = .021); therefore, nonparametric-independent 2-sample test was employed. Accordingly, IDH1 mutant group mean age (39.2 ± 12.1) was not significantly different (*p* = .489) from the IDHI-wild group mean age (41.4 ± 8.9). In contrast, the MMSE performance on the concentration-calculation subtest was found significantly different (*p* < .01) between IDH1 mutant and IDH1 wild groups.

#### Neuropsychological tests and tumor parameters.

—In the total sample, dominant hemisphere tumor patients scored lower in the MMSE concentration calculation and in DS backward subtests than their nondominant hemisphere counterparts, but this difference did not reach statistical significance (*p* = .08 and *p* = .61, respectively). TMT B scores were also comparable between the two hemispheres (*p* = .23). Laterality comparison was not employed for COWAT as the test was administrated only in patients with dominant hemisphere HGG.

Regarding tumor localization, ANOVA Kruskal–Wallis test showed that mean performance on MMSE concentration subset (*p* = .23), DS backwards (*p* = .11), TMT B (*p* = .08), and COWAT phonemic (*p* = .34) was not significantly different across frontal, temporal, and parietal lobes. The occipital lobe was not included in the analysis as we encountered only 1 patient with occipital HGG. On the contrary, MMSE concentration-calculation subset (*r* = −397, *p* < .01), DS backwards (*r* = −4.23, *p* < .01), TMT B (*r* = −6.12, *p* < .01), and COWAT phonemic (*r* = −5.45, *p* < .01), mean performances showed statistically significant inverse correlations with tumor volume in the total sample. In the same line, comparisons between tumor grade groups on MMSE concentration-calculation, DS backwards, TMT B, and COWAT phonemic subtests showed a statistically significant difference (*p* ≤ .01), with grade IV group performing poorer than the grade III one.

When we split the data according to IDH1 mutation status, there was no statistically significant difference in MMSE concentration-calculation, TMT B, and DS backwards means between the left-hemisphere and right-hemisphere tumor patients neither for the IDH1 mutant group (*p* = .10, *p* = .45, and *p* = .31, respectively) nor for the IDH1 wild one (*p* = .08, *p* = .20, and *p* = .28, respectively). Similarly, tumor lobe localization had no significant effect on MMSE concentration-calculation, DS backwards, TMT B, and COWAT phonemic score means, neither in the IDH1 mutant (*p* = .39, *p* = .20, *p* = .09, and 0.11, respectively) nor in the IDH1 wild-type (*p* = .22, *p* = .32, *p* = .17, and *p* = .38, respectively) group. Tumor volume, on the other hand, showed an inverse correlation with MMSE concentration-calculation subtest performance (*r* = −.401, *p* < .01), TMT B (*r* = −.328, *p* < .01) and COWAT phonemic score (*r* = −.599, *p* < .01); however, only for the IDH1 wild-type group and not for the IDH1 mutant one (*r* = .060, *p* = .791; *r* = −.023, *p* = .345, and *r* =.110, *p* = .89). Finally, concerning the effect of tumor grade on neuropsychological testing for each IDH1 mutation group, our results showed that there was no statistical significance in MMSE concentration-calculation, DS backwards, TMT B, and COWAT phonemic scores (*p* > .05) between the 2 IDH1 mutation subgroups of grade IV tumor patients. On the contrary, for the grade III group, we found a statistically significant difference in TMT B (*p* < .01) and DS backwards (*p* < .01) between IDH1 subgroups with the mutant one to outperform the IDH1wild one.

### ANCOVA Analysis

ANCOVA analyses with IDH1 mutation status as an independent factor and each of the cognitive measures of interest (TMT B, DS backwards, COWAT phonemic subset, and MMSE calculation-concentration subset) as dependent variables with age, gender, tumor volume, and grade as covariates were also conducted in order to assess significance survival. Analysis for TMT B showed a significant difference (*F*[(1, 146] = 28.9, *p* ≤ .01), between the 2 IDH1 groups with IDH1 mutant group to outperform the IDH1 wild one. Similarly, a significant difference (*F*[1, 146] = 5.64, *p* = .02) was found for DS backward measure with IDH1 wild group to perform poorer than the IDH1 mutant one. IDH1 status had also significant effect on COWAT phonemic subset (*F*[1, 146] = 6.47, *p* = .01)] and a marginal one in MMSE calculation-attention subtest (*F*[1, 146] = 2.38, *p* = .05).

## Discussion

In the present study, we sought to investigate the association between NCF, executive functions, in particular, and IDH1 genetic mutation status in HGGs in order to shed light on the role that tumor proliferation rate may have on NCF impairment often seen in HGGs. Executive functions were found impaired in our overall sample, highlighting the well-known notion that a glial tumor can have a negative impact on higher cerebral processing.^18,19,24–26^ However, when we examined the relationship between IDH1 mutation status and NCF in HGGs, we observed that patients with IDH1 mutant-type tumor exhibited less severe cognitive deficits in comparison to patients harboring IDH1 wild-type one.

Demographical parameters such as patient’s age would be a confounding variable in our results. Indeed, according to our findings, age was significantly higher in the IDH1 wild group compared to IDH1 mutant one, and correlated in parallel with the MMSE concentration-calculation scores. This, however, was observed only in the IDH1 wild group indicating that age may not be the primary factor affecting NCF in our clinical sample. In parallel, comparison analyses between the two—age matched—subgroups of our cohort showed that MMSE concentration-calculation performance was still significantly poorer in IDH1 wild group as compared to IDH1-mutant one. In addition, for the measurement of working memory (DS), verbal fluency (COWAT), and complex attention (TMTB) functions, we employed standardized age scores; therefore, we consider our results unaffected by age. Taking all the above into account, our findings demonstrate that the age of patients was not the principal factor that NCF differences seen between the two IDH1 mutation status groups can be attributed to.

Lesion characteristic such as tumor size is a well-known parameter that may affect the clinical status of tumor patients.^13^ However, our findings suggest that tumor volume may not be the principal factor affecting NCF negatively. Although patients with larger tumors were presented with greater executive function impairment in our total sample, that was not the case when we accounted for IDH1 mutation status. Tumor size was comparable between the 2 IDH1 mutation status groups, and although we found an inverse association between the tests of MMSE concentration-calculation, verbal fluency and complex attention, and tumor size for IDH1 wild-type group, a similar association was not demonstrable for IDH1 mutant-type group. In fact, the tumor size mean was slightly larger in IDH1 mutant group; if the lesion size was the main factor affecting executive functions’ status, one would expect IDH1 mutant group to show more impairment compared to IDH1 wild-type one. However, our findings demonstrated the opposite pattern. It is noteworthy to mention that our results are in line with the ones of Wefel et al.^15^ reporting similar findings on the relationship between NCF and lesion size.

Apart from size, tumor location may play a role in the differences of cognitive impairment seen in patients with HGG glioma. In line with other studies,^27^ the majority of tumors in our overall cohort were located in the frontal and temporal lobes; this finding was consistent even when we split patients according to IDH1 mutation status, with both IDH1 mutant and IDH1 wild-type groups to show similar rates of tumor localization. In addition, the distribution of tumors across hemispheres was similar for both IDH1 mutation status groups. Therefore, our findings suggest that tumor location alone would not sufficiently explain the differences seen in our patients’ executive function status.

Traditional III/IV grading classification could be potentially the primary factor for the differences we observed in our patients’ cognitive performance as, consistently with the literature, the majority of grade III gliomas were IDH1-mutant-type ones and the majority of grade IV gliomas were IDH1 wild-type ones. Indeed, according to our results and in line with previous research,^15^ patients with grade IV malignancy performed significantly worse on all the neuropsychological tests than their grade III counterparts. Due to the intercorrelation between malignant grading and IDH1 mutation status, no study has previously sought to investigate the effect of IDH1 mutation status within grade III and IV separately. In the present study, we, first in the literature, attempted to address this issue and we found a significant difference in NCF in patients harboring a grade III glioma, with those diagnosed with mutant ones to outperform their wild counterparts on some of the executive functions, complex attention (TMT B), and working memory (DS backwards) namely. The lack of the aforementioned difference in grade IV glioma group would be possibly attributed to the small percentage (7%) of grade IV IDH1 mutant-type glioma we encountered in our sample, consistent with the literature. Accordingly, our findings indicate that baseline neurocognitive status may largely depend on the IDH1 mutation status rather than on the simplified tumor III and IV grading per se. However, future studies with larger cohorts of IDH1 mutant status subcategories within grade III and especially within grade IV are needed in order to extend our results.

By combining the differences in executive function impairment by IDH1 mutation status, we observed in the present study and the fact that tumor location alone would not sufficiently explain these differences. Our results support that tumor proliferation kinetics may indeed have a more profound impact on NCF status than tumor size alone. Thus, our findings support indirectly the notion held by other studies^15^ that lesion momentum has a great impact on NCF, especially in executive functions, with IDH1 mutant-type tumors to allow for more neuroplasticity and to induce less cognitive impairment than IDH1 wild tumors.

Previous research using magnetoencephalography has also shown differences in global functional connectivity between IDH subgroups that were correlated to patients’ neurocognitive status with IDH wild-type glioma patients showing poorer performance than the IDH mutant ones.^28^ According to the authors, these differences may indeed mirror the impact of the tumor growth rate on brain’s global connectivity. Another study,^29^aiming to investigate the effect of IDH1 mutation on the structural connectome, has shown that wild-type tumor patients demonstrate lower network efficiency and more frequent cognitive impairment than mutant ones. The authors supported that cognitive reserve appeared to mediate the inverse relationship between network efficiency and cognitive status in IDH1 mutant group, indicating a significant amount of neuroplasticity in these patients. Overall, the literature indicates that differences in neurocognitive status between IDH1 subgroups may reflect the differences in neuroplasticity, that is, the brain’s ability to adapt to a tumor, with IDH1-wt gliomas to allow a limited one. On the other hand, one could assume that the *IDH1* mutation may affect directly NCF; however, IDH1 mutations result in the production of 2-hydroxyglutarate, which may lead to neurodegeneration and neurological cognitive deficits, as in D-2-hydroxyglutaric aciduria, a neurometabolic disease.^30^Thus, it is more plausible that the favorable cognitive status in *IDH1* mutant gliomas is a result by associated molecular genetic characteristics, which may, in turn, lead to increased associated metabolic changes, less growth velocity, and thus to greater plasticity of the adjacent brain tissue.

In our methodology, only IDH1 R132H IDH1 was included in our analysis, consisting thus a limitation in the present study. IDH2 mutation has been detected in 0.9% of anaplastic astrocytomas^7^ and noncanonical IDH1 mutations—although have not been associated with different prognostic values from the canonical ones—account for 7.9% of anaplastic gliomas.^31^Thus, in our cohort, we may have missed 3 cases with these mutations, if a respective analysis was conducted. Although IDH2 and noncanonical IDH1 mutations are seen rarely in AA, future studies should address a potential relationship between executive functions and IDH2 and noncanonical IDH1 mutations. Future researchers should also investigate whether new—according to the 2021 WHO CNS tumor classification^32^ gene and molecular alterations (ATRX, TP53, CDKN2A/B for astrocytoma IDH mutant and TERT promoter, chromosomes 7/10, and EGFR for GBM IDH wild) influence cognitive performance through mechanisms that include perturbation of neuronal communication.

Overall, our study provides preliminary evidence for the association of neurocognition—of executive functions in particular—with IDHI1 genetic mutation status in patients harboring an HGG, after accounting for other factors that could potentially have an impact on patients’ neuropsychological status. Our outcomes stress the need for the incorporation of neurocognitive assessment in preoperation/treatment workup in patients with HGGs. Given the strong associations between IDH1 status and NCF, but also between IDH1 status and overall survival in patients with AA and GBM, baseline neurocognitive status would add in the future—along with other factors—a prognostic tool which, in turn, could assist physicians in selecting the best treatment plan.
